# CAR T-Cell Therapy Predictive Response Markers in Diffuse Large B-Cell Lymphoma and Therapeutic Options After CART19 Failure

**DOI:** 10.3389/fimmu.2022.904497

**Published:** 2022-07-06

**Authors:** Ana Carolina Caballero, Laura Escribà-Garcia, Carmen Alvarez-Fernández, Javier Briones

**Affiliations:** ^1^ Hematology Service, Hospital de la Santa Creu i Sant Pau, Barcelona, Spain; ^2^ Laboratory of Experimental Hematology-IIB, Institut Recerca Hospital de la Santa Creu i Sant Pau, Barcelona, Spain; ^3^ Campus Sant Pau, Josep Carreras Leukemia Research Institute, Barcelona, Spain; ^4^ Autonomous University of Barcelona, Barcelona, Spain

**Keywords:** CAR-T, DLBCL, lymphoma, antibodies, bispecific

## Abstract

Immunotherapy with T cells genetically modified with chimeric antigen receptors (CARs) has shown significant clinical efficacy in patients with relapsed/refractory B-cell lymphoma. Nevertheless, more than 50% of treated patients do not benefit from such therapy due to either absence of response or further relapse. Elucidation of clinical and biological features that would predict clinical response to CART19 therapy is of paramount importance and eventually may allow for selection of those patients with greater chances of response. In the last 5 years, significant clinical experience has been obtained in the treatment of diffuse large B-cell lymphoma (DLBCL) patients with CAR19 T cells, and major advances have been made on the understanding of CART19 efficacy mechanisms. In this review, we discuss clinical and tumor features associated with response to CART19 in DLBCL patients as well as the impact of biological features of the infusion CART19 product on the clinical response. Prognosis of DLBCL patients that fail CART19 is poor and therapeutic approaches with new drugs are also discussed.

## Introduction

B-cell non-Hodgkin lymphomas (B-NHL) comprised some 544,000 new cases/year and caused 260,000 deaths worldwide in 2020 (1). Diffuse large B-cell lymphoma (DLBCL) is the most common subtype, accounting for 30%–40% of all newly diagnosed lymphomas in the world. DLBCL has an aggressive behavior and needs rapid treatment (2). First line of treatment is usually composed of repeated cycles of chemoimmunotherapy with rituximab combined with cyclophosphamide (Cy), doxorubicin, vincristine, and prednisone (R-CHOP) that allows to achieve long-term disease remissions in 60% of cases (2, 3). Forty percent remains unresponsive, either due to primary refractoriness (10%–15%) or relapse after having achieved an initial complete response (20%–25%) (4).

Overall survival (OS) in relapsed or refractory (R/R) subgroup patients is poor. High-dose chemotherapy followed by autologous stem cell transplantation (ASCT) is the standard of care (SOC) in this situation for patients considered suitable for that treatment. About a half of R/R DLBCL patients are not eligible for intensive approach due to advanced age or comorbidities. There is no salvage regimen that has proven to be superior with overall response rates (ORRs) of 40%–50% and only ~20% of complete responses (5). This means that only up to 50% of patients who are potentially candidates for intensive treatment would reach a required response to proceed to ASCT (2, 4–6). Among patients who received ASCT, less than 50% would be disease-free after 5 years (5). Refractory patients, defined as DLBCL that did not achieve objective response or relapsed ≤12 months after treatment, remain the greatest challenge. For these patients, current salvage treatments yield ORR approximately 26% with less than 10% CRs, and their median OS was reported to be as short as 6.3 months in some studies (4).

**Figure 1:**
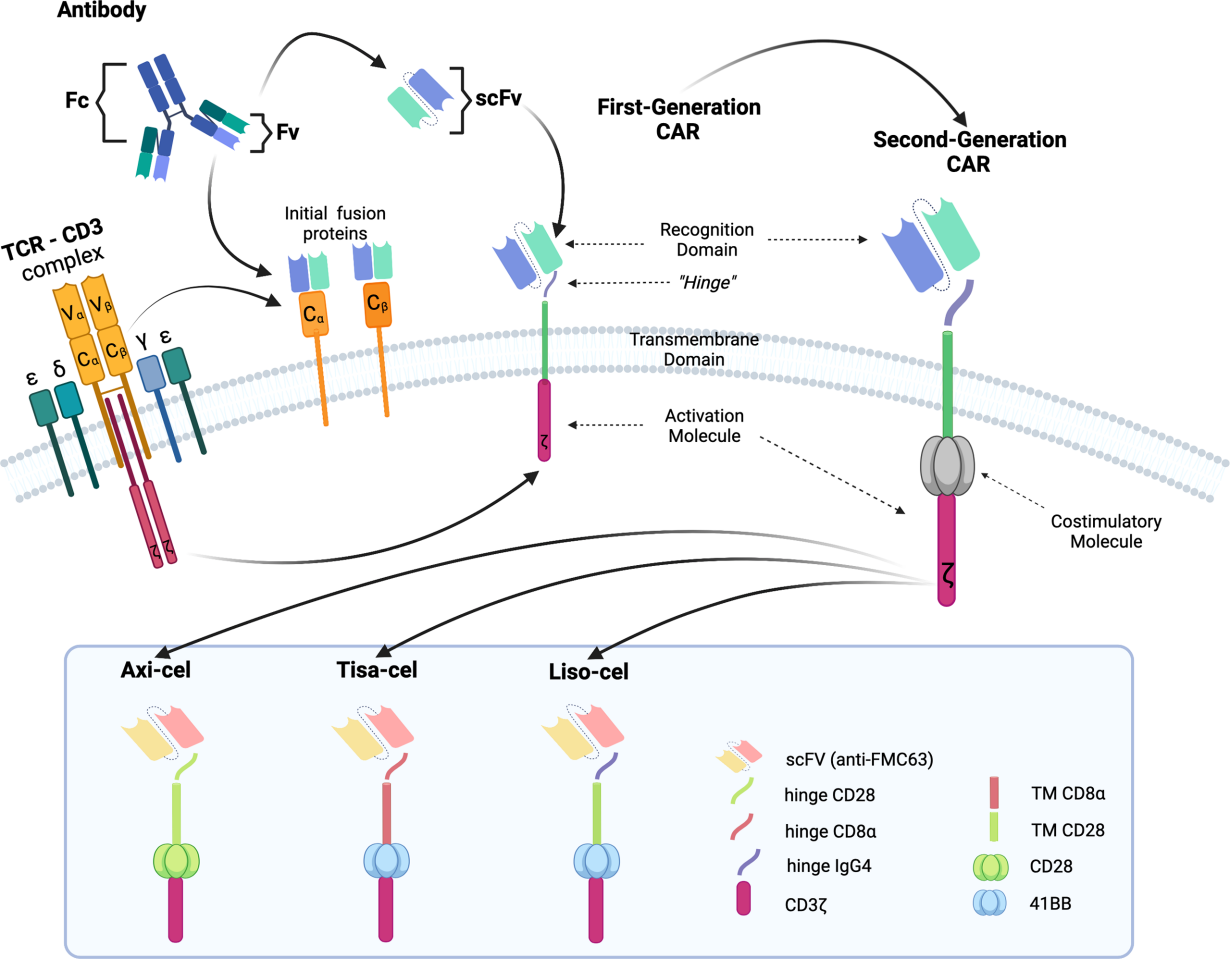
Illustration of the evolution of chimeric receptors design. Fragments from the native TCR-CD3 complex and antibodies were joined into fusion proteins that were improved to reach the basic structure of the second-generation CARs, which were successfully brought to the clinic. At the bottom, the figure shows the critical differences between the three FDA-approved second-generation CAR constructs.

The poor outcomes shown by patients with R/R DLBCL constitute an unmet medical need and have allowed adoptive cell therapy (ACT) to change the paradigm of lymphoma treatment. Initially, ACT sought to sensitize and expand tumor-reactive T-cell *in vivo* and direct isolation of tumor-infiltrating lymphocytes (TILs) has been tested in multiple solid tumor studies with some durable responses, particularly in melanoma (7). ACT has evolved to increase efficacy and specificity. In late 1980s, first-generation chimeric antigen receptors (CARs) were described. Initially, they were constructed by fusing T-cell receptor (TCR) constant domain (segments α or β chains) and the variable region of an antibody, both the heavy chain and the light chain (8). Next, recognition domain was simplified into a single-chain peptide structure derived from heavy and light chain variable region of a specific immunoglobulin (scFv). The scFv was fused with ζ chain of the TCR CD3 complex, and thus a first-generation CAR was generated. CAR T cells containing CD3ζ demonstrated antitumor activity *in vitro* but exhibited limited *in vivo* antitumor effect, anergy, and lack of expansion (9, 10). In late 1990s, second-generation CARs were built by adding a costimulatory domain (CD28) to the initial first-generation CAR construct (**Figure 1**). These changes were based on the knowledge of TCR function to optimize *in vivo* expansion of activated T cells (11). Second-generation CAR directed against CD19 (CAR19) was shown to be effective in B acute lymphoblastic leukemia (B-ALL) animal models (12). Early studies with CAR20 T cells showed limited efficacy in patients with B-NHL, presumably because they were done with a first-generation CAR (13).

Finally, CAR T cells moved into the clinical setting when treating a patient with refractory follicular lymphoma who reached partial response (PR) after CART19 therapy (14). Then, a few academic studies published their earliest clinical experiences with CAR19 T cells, paving the way for the development of CART19 clinical trials for patients with B-NHL (15–21).

## Overview of Clinical Trials in DLBCL With Clinically Approved CART19

Clinical trials with CART19 therapy have shown great efficacy in heavily pretreated patients with DLBCL, high-grade B lymphoma, and primary mediastinal B-cell lymphoma (PMBCL). Outstanding results in academic studies led to the clinical development and subsequent approval by regulatory agencies of three different CAR19 T-cell products (**Figure 1**).

Axicabtagene ciloleucel (Axi-cel, Yescarta) is a CAR19 T-cell product originally designed by researchers at the National Cancer Institute (15, 16, 22) and later developed by Kite-Gilead. Autologous peripheral blood mononuclear cells (PBMCs) from fresh leukapheresis are the starting material. PBMCs are genetically modified by gamma retroviral vector to express a CAR. Axi-cel uses a second-generation CAR with anti-CD19 scFv as recognition domain, CD3 ζ as activation, and CD28 as costimulation domain. Axi-cel has been approved to treat patients with R/R DLBCL, PMBCL, or DLBCL transformed from follicular lymphoma (FL), R/R to at least two previous lines of treatment. The approval was derived from the results of ZUMA-1, a phase 1/2 clinical trial that treated 101 patients (77 with DLBCL and 24 with PMBCL) with a single dose of 2 × 10^6^ CAR19^+^T cells/kg. All patients received lymphodepletion (LD) regimen with Cy 500 mg/m^2^/day, and fludarabine (Flu) 30 mg/m^2^/day for 3 days before infusion. The median age of treated patients was 58 years (range: 23–76). Most of them had stage III or IV (85%), primary refractory disease comprised 2% and another 77% was refractory to second line or subsequent. Twenty-one percent of patients had history of recurrent disease after ASCT. Bridging therapy before CART19 infusion was not allowed. Cytokine release syndrome (CRS) occurred in 93% of patients and 13% of them were grade 3 or higher. Neurologic events (ICANS) occurred in 64% of cases and 23% were grade ≥ 3. ORR was 82% with 54% CRs. The median duration of response (DOR) was 11.1 months, and after a median follow-up of 27 months, the two-year progression-free survival (PFS) was 41% and OS was 50% (23).

Tisagenlecleucel (Tisa-cel, Kymriah) is a CAR19 T-cell product derived from studies at the University of Pennsylvania followed by clinical development by Novartis (17–19). T cells are isolated from autologous cryopreserved leukapheresis, as starting material. Isolated T cells are transduced with a lentiviral vector encoding a second-generation CAR costimulated with 4-1BB (CD137), targeting CD19 and having CD3 ζ as activation domain. Tisa-cel has been approved for the treatment of R/R DLBCL after at least two previous lines of treatment. JULIET, a phase 2 clinical trial, was the pivotal study and included 111 patients (88 DLBCL and 21 DLBCL transformed from FL) who were treated with a single infusion of Tisa-cel. The median dose administered was 3 × 10^8^ CAR19^+^T cells (range: 0.6–6 × 10^8^). Cy 250 mg/m^2^/day and Flu 25 mg/m^2^/day for 3 days before infusion was used as LD regimen. The median age of the included patients was 56 years (range: 22–76), stage III or IV was in 76% of cases, 55% of patients were refractory to previous line, and 49% had received ASCT. Most of the patients (92%) received bridging therapy before CART19 administration. CRS was observed in 56% of patients, but only 22% of grade ≥3 was detected. ICANS was less common (21%) and less severe (12% grade ≥3). ORR was 52% with 40% CR. PFS at 12 months was 35% for all patients (24).

Lisocabtagene maraleucel (Liso-cel) is a second-generation CAR19 T-cell product originally designed and formulated by investigators at the Fred Hutchinson Cancer Research Center and then continued by Juno and Bristol-Myers Squibb. Liso-cel is generated from fresh autologous leukapheresis. CD4^+^ and CD8^+^ T cells are selected and separated into two different fractions to be cultured in parallel and are genetically modified by lentiviral vector encoding a second-generation CAR19 costimulated with 4-1BB (CD137) and an additional sequence encoding a truncated epidermal growth factor receptor (EGFRt) to guarantee identification of transduced T cells. The transduced CD4^+^ and CD8^+^ T-cell fractions are mixed in a 1:1 ratio to obtain the final formulation (20, 21). Liso-cel is approved for the treatment of R/R DLBCL, PMBCL, and FL grade 3B (FL3B) after at least two prior therapies. TRANSCEND NHL001 study led to FDA approval for Liso-cel. It was a phase 1/2 clinical trial and included 269 patients (173 DLBCL, 78 DLBCL transformed from indolent lymphoma, and 15 PMBCL). Median age was 63 years (range: 54–70), 67% of patients had refractory disease, and 33% patients had previously received ASCT. Bridging therapy was administered to 59% of patients. All patients received LD regimen with Cy 300 mg/m^2^/day and Flu 30 mg/m^2^/day for 3 days before infusion. CRS was observed in 42% of cases, but only 2% developed severe CRS (≥3 grade). ICANS was detected in 30% of cases and 10% of them were higher than grade 3. Phase 1 sequentially evaluated three doses: 50 × 10^6^ CAR19^+^ (dose 1), 100 × 10^6^ CAR19^+^ (dose 2) and 150 × 10^6^ CAR19^+^ (dose 3). Dose 2 (100 × 10^6^ CAR19^+^) was selected for phase 2 to test efficacy. The ORR was 73% with 53% of CRs. PFS at 12 months was 48% with an OS of 58% for the overall patient population (25).

In summary, CAR T-cell therapy progressively evolved in the last 2 decades to finally breakthrough the SOC treatment for R/R DLBCL and other B-cell neoplasms. A few academic research groups and pharmaceutical companies brought their own CAR19 T-cell product into the practice through clinical trials, which were different in design, but led to approval and commercialization of all three products: Axi-cel, Tisa-cel, and Liso-cel. Main differences in relevant features among these pivotal clinical trials are summarized in **Table 1**. Different primary endpoints, evaluations, and statistical analysis in these studies were added to distinct intrinsic features of the CAR19 T-cell product: starting material, dose, T-cell composition, culture conditions, and constructs design. Despite this, surprisingly similar efficacy was seen among all three products with remissions approximately 50% in this previously incurable patient population, although differences in toxicity were noticed in the clinical trials.

**Table 1:** Pivotal Clinical Trials with CART19 Therapy for B-NHL: Comparison of Relevant Features Among Studies.

Clinical trial	ZUMA-1	JULIET	Transcend
Product name	Axicabtagene ciloleucel	Tisagenlecleucel	Lisocabtagene maraleucel
Co-stimulatory domain	CD28	4-1BB	4-1BB
Leukapheresis	Fresh	Cryopreserved	Fresh
Starting material	PBMCs	Selected T cells	Sorted CD4^+^ and CD8^+^ fractions
Transduction	Retroviral	Lentiviral	Lentiviral
Final composition	Bulk T cells	Bulk T cells	Defined ratio CD4^+^:CD8^+^ 1:1
Patients enrolled, n	111	165	344
Infused patients, n	101	111	269
Study population	DLBCL PMBCL tFL	DLBCL tFL	DLBCL DLBCL transformed from indolent lymphoma PMBCL FL grade 3B
Bridging therapy (% patients)	Not allowed	Allowed (92%)	Allowed (59%)
Lymphodepleting therapy	Cy 500 mg/m^2^/day + Flu 30 mg/m^2^/day 3 days	Cy 250 mg/m^2^/day + Flu 25 mg/m^2^/day 3 days	Cy 500 mg/m^2^/day + Flu 30 mg/m^2^/day 3 days
Stage III/IV	85%	76%	NR
>3 prior lines	69%	52%	51%
Refractory disease	77%	55%	67%
Previous ASCT	21%	49%	33%
Cell origin			
*Germinal center B-cell–like subtype*	49%	57%	NR
*Activated B-cell–like subtype*	17%	41%	NR
High-grade B-cell lymphoma	NR	27%	13%
Dose	2 × 10^6^ CAR T cells/kg	Median dose: 3 × 10^8^ CAR T cells/kg (0.1 × 10^8^–6 × 10^8^)	1 × 10^8^ CAR T cells (50 × 10^6^ CD8^+^ + 50 × 10^6^ CD4^+^ CAR T cells)

DLBCL, diffuse large B cell lymphoma; PMBCL, primary mediastinal B cell lymphoma; tFL, transformed follicular lymphoma; NR, not reported.

Although CART19 therapy has been successful in treating and probably curing some patients with R/R DLBCL, the fact is that more than half of them remain refractory or relapse after CART19. There is room for improvement and the treatment of multirefractory patients is increasingly challenging. In-depth knowledge of current outcomes and associated clinical or biological characteristics of patients and product properties that influence therapy success is essential to guide treatment, modulate expectations, and decide on the most effective salvage therapy after CART19 failure.

## Clinical Outcome After CART19 Therapy Is Influenced by Patient and Disease Features

All published data agree that CART19 therapy improved R/R DLBCL clinical outcomes and OS; however, identifying the subgroup of patients who would certainly benefit from CART19 remains a challenge. Patient selection criteria in clinical trials are usually homogeneous and comorbidities are often underrepresented, making it difficult to identify differential baseline characteristics that may influence clinical outcome. Thus, the pivotal studies of Tisa-cel and Axi-cel agreed that there were no clinical covariates predictive of efficacy (23, 24). In contrast, Liso-cel pivotal trial, which was the largest and the most variable in terms of diagnoses (including R/R DLBCL, R/R PMBCL, transformed DLBCL arising from indolent histologies other than FL and FL3B), showed that DOR and PFS in patients with PMBCL and DLBCL transformed from FL were longer than for other subtypes and that bridging therapy was associated with lower efficacy among included patients, likely reflecting a selection bias (25).

Data from a retrospective study, which analyzed baseline patient characteristics to evaluate whether classical prognosis factors could distinguish patients most likely to achieve disease remission, showed associations between achieving CR at 12 months after Axi-cel treatment and no need for bridging therapy (26). The need for bridging therapy reflects higher tumor burden (TB) or more rapidly progressive disease and thus emerges as a negative prognostic feature. Other patient-dependent features, such as age (≥60years), ECOG 0/1, and normal lactate dehydrogenase (LDH) at the time of conditioning, were also described to be associated to CRs after Axi-cel treatment. These are important findings considering that attaining CR is the most important goal after treatment of aggressive lymphomas (26). Performance status, no need for bridging therapy, and normal LDH values before CAR19 T-cell infusion were also associated to longer PFS (26–28). Finally, a recent study that evaluated international prognostic index (IPI) and age-adjusted IPI, two widely used indices to identify DLBCL patients with higher probability of survival following frontline therapy, found an association with them and PFS after CART19 treatment (29).

Several tumor-related prognosis factors routinely analyzed in DLBCL (i.e., cell of origin, myc, and bcl-2 rearrangements) did not prove to be informative of sensitivity to CART19 therapy. Preliminary studies on the role of such tumor features in patients treated with CART19 in different trials did not find a strong impact on CART19 tumor resistance, with comparable clinical responses observed across all these mentioned subgroups (24, 30). P53 alterations are described markers of poor outcome and treatment resistance in DLBCL (31). A recent study found that DLBCL patients with tumors harboring P53 alterations had lower responses and survival after CART19 therapies, particularly following a 4-1BB costimulated second-generation CART19 (32). DLBCLs with P53 were found to have more frequently downregulation of genes related to interferon signaling pathway, which may contribute to further inhibition of CAR19 T cells. However, this is in contrast to the findings of a previous study showing a correlation with a high interferon-related gene expression and lack of durable response after Axi-cel treatment (33). Further studies are needed to establish the role of this critical signaling pathway on the resistance of DLBCL to CART19 therapy.

Even though some studies conclude that distinct LD regimens do not affect clinical outcomes (34), it has been shown that LD plays a role in both T-cell kinetics of expansion and clinical outcome in B-cell malignancies. Elimination of regulatory T cells (T_regs_) and improved function of transferred T cells by increasing the availability of homeostatic cytokines are among the proposed mechanisms that explain the positive impact of LD on the efficacy of adoptive therapy. Interleukin-7 (IL-7) and interleukin-15 (IL-15) have supporting roles in the survival and proliferation of adoptively transferred T cells and have been proved to be critical to enhance adoptive T-cell antitumor effect *in vivo* (35). Flu combined with Cy represents the most frequently LD regimen used and enhances CD4^+^ and CD8^+^ proliferation and persistence of 41BB-CAR19 T cells in R/R ALL patients (36). Furthermore, the intensity of the LD regimen may positively impact on the outcome after CART therapy, as it has been shown after adoptive therapy of TILs (37). Elevated levels of IL-7 and monocyte chemoattractant protein-1 (MCP-1) were associated with superior expansion and better PFS in patients with B-NHL treated with CART19 and, interestingly, this favorable cytokine profile was found more frequently in those patients receiving higher doses of that combo (28). In addition, lymphoma patients who achieve CR developed a greater increase of serum IL-15 levels after Flu/Cy LD and a greater area under the curve (AUC) of CAR19 T cells up to day +14 after infusion (38). Recent studies found that optimizing Flu exposure may have a relevant impact in PFS and OS of B-ALL R/R patients after CART19 (39, 40). Examination of cumulative Flu exposure suggest that an AUC ≥ 14 mg*h/L is associated with a lower proportion of CD19^+^ relapses, lower cumulative incidence of B-cell recovery after CART19, and higher CAR19 T-cell expansion (39). In agreement with these data, another study in B-ALL found that a suboptimal Flu exposure (defined as AUC ≤ 13.8 mg*h/L) was associated with increased risk of relapse and loss of B-cell aplasia. Furthermore, Flu exposure was noted to affect OS in patients with high pre-infusion TB (40). These data indicate that adjustment of Flu dose may have a relevant impact on CART19 efficacy in patients with B-ALL, which should be prospectively studied in patients with DLBCL. Overall, these data suggest the environment (influenced by conditioning) into which CAR T cells enter upon infusion and modulate CAR T-cell functionality, which may translate into improved treatment outcomes. Other markers related to a pro-inflammatory setting have been shown to have an impact on clinical results. Thus, elevated LDH and IL-6 before treatment have been associated to lower durable response rates, while high pretreatment CRP, ferritin, and LDH levels have been associated with lower CAR19 T-cell expansion (41, 42).

Higher peak and longer persistence of CAR19 T cells were shown to determine a better PFS in R/R ALL patients (36). Persistence of CAR19 T cells evaluated by flow cytometry was found to be longer in patients with DLBCL R/R who achieved response after treatment with Tisa-cel (43). Greater CAR19 T-cell peak and AUC in the first 28 days after infusion have been reported to be associated with objective and durable response in lymphoma patients treated with Axi-cel (30, 41, 44). Furthermore, the Liso-cel pivotal clinical trial evidenced a median maximum expansion (Cmax) and AUC significantly higher in responders compared to non-responders (25). Besides, further analyses revealed the value of estimating the number of CAR19 T cells per unit of blood volume, which seems to be even more informative than the number of CAR19 gene copies per microgram of host DNA, to predict response (41). Overall, data from different clinical studies demonstrate that a high-peak expansion of CAR19 T cells in peripheral blood correlates with a durable response in B-cell neoplasms (25, 30, 44, 45).

In addition to the achievement of a favorable cytokine profile in the host, antigen exposure on tumor cells can also modulate CAR T-cell expansion. As a proof of concept, a superior expansion of CAR19 T cells has been detected in patients with high TB in R/R B-ALL. However, comparison between these studies is hampered by the fact that the definition of high TB was highly heterogeneous (36, 46–48). Although similar patterns of CAR19 T cells increase and biexponential decline were observed in DLBCL and B-ALL, CAR19 T-cell peak in peripheral blood is lower in DLBCL and a number of studies showed no apparent relationship between TB and expansion (24, 43, 49). In line with this, there was a remarkable finding of sustained CAR19 T-cell expansion and long-term CR in DLBCL patients treated with CART19 and without detectable disease (according to FDG-PET/CT) at the time of infusion (50). Furthermore, the limited clinical data available indicates that patients with low intensity of CD19 expression in tumor samples did not have inferior responses (24, 30, 43).

Another feature associated to CART19 efficacy is tumor volume. DLBCL patients with bulky disease have inferior clinical outcomes. Recently, measurements of pretreatment FDG-PET/CT images have allowed to estimate TB more accurately and a greater metabolic tumor volume (MTV) has been proposed as a new predictor of lower PFS and OS. However, heterogeneous cutoffs for high or low TB were used in these studies and hence formal clinical validation is lacking (51–53). Retrospective studies tried to extend baseline MTV evaluation to DLBCL patients treated with CAR19 T cells and concluded that patients with a low MTV have superior OS and PFS (41, 42, 54, 55). Preliminary data suggest that MTV and average standardized uptake value evaluated early after therapy are independent risk factors associated to OS and PFS (55). Nonetheless, other studies found discrepant results with no significant differences in clinical outcome according to MTV (56), highlighting the differences in type of measurement, methodological aspects, and definitions. Overall, future research should also include tumor debulking prior to CART therapy, as current data suggests that patients with a lower TB at the time of CAR19 T-cell infusion are more likely to be cured (57).

New diagnostic techniques that emerge as promising predictors of relapse in DLBCL converge with the introduction of new therapies. Circulating tumor DNA (ctDNA), released from apoptotic and/or necrotic tumor cells, is an emerging prognostic biomarker for lymphoma. Next-generation sequencing-based assays that utilize immunoglobulin gene V(D)J rearrangements as a marker of clonality for detecting cancer cells have been applied to patients with lymphoid malignancies, and surveillance ctDNA has been shown to identify patients at risk of relapse before clinical evidence of disease after first-line treatment (58–60). In relation to this, a prospective study explored the prognostic value of ctDNA after Axi-cel treatment. In this study, high ctDNA concentrations before treatment were associated with progression after Axi-cel infusion, while non-detectable ctDNA at day 28 after treatment was correlated with longer PFS (61). In addition, preliminary data on profiling genomic alterations in tumor DLBCL samples suggest that the median numbers of plasma ctDNA mutations after CART19 treatment in patients who remained in CR and patients who relapse were extremely different (62). Taken together, ctDNA clonotype and tumor mutations surveillance can be useful to determine prognosis and even detect specific targets for designing treatment strategies after CART19 failure.

## CAR T-Cell Product Composition: The Importance of Less Differentiated Memory T Cells

### Early-Memory T-Cell Subsets and CART19 Efficacy

Following antigenic stimulation, “naïve” T cells (T_N_) proliferate and differentiate into memory T cells (T_M_) and effector T cells (T_EF_). T_M_ lymphocytes play a fundamental role in the immune response against cancer. In humans, the T_M_ cell population is heterogeneous with respect to phenotype and function but can be divided into two groups (63): central memory T cells (T_CM_), which express CD45R0, CD62L, and CCR7; and effector memory T cells (T_EM_) lacking CD62L and CCR7. Several studies in animal models of cancer have shown that T_CM_ cells have a superior antitumor effect than differentiated T_EM_ and T_EF_ cells (64). A subtype of memory T cells, the memory stem T lymphocytes (T_SCM_), has been identified in humans with distinct gene expression and functional attributes to other T-cell subsets (65, 66). These cells represent 2%–3% of the entire T-cell population in peripheral blood from healthy individuals. T_SCM_ cells are characterized by expressing naïve and memory markers such as CD45RA, CD62L, and CCR7 but, unlike T_N_ cells, they express high levels of CD95, CXCR3, CD58, and IL2Rβ. (65, 67) T_SCM_ cells have the ability to self-renew and to generate all the progeny of memory (T_CM_ and T_EM_) and effector (T_EF_) T cells (66, 68). Compared to central memory and effector T cells, this unique population has a higher proliferative and persistence capacity *in vivo* and, importantly, a superior antitumor effect in animal models of cancer (69); this attribute also holds true for those T_SCM_ gene-modified with an antigen-specific CAR; thus, adoptive transfers of sorted memory T-cell subsets (e.g., T_SCM_, T_CM_, and T_EM_) transduced with a mesothelin-redirected CAR into a xenogeneic model of human mesothelioma demonstrated a superior antitumor effect of the T_SCM_ subset, which was also associated to enhanced proliferative capacity and persistence of CAR T cells (65, 70).

It is well established that cellular CART products infused into patients are quite heterogeneous, related to T-cell subset composition as well as exhaustion, all of these being tumor dependent (i.e., T cells from CLL patients may be more differentiated and exhausted than in other B-NHL). Collectively, these differences may contribute significantly to the efficacy of the CART19 therapy. Furthermore, patients with DLBCL have substantial differences in their frequencies of peripheral blood T-cell subsets (naïve, T_CM_, or T_EM_) (20, 21), due to factors such as age and chemotherapy regimens received (71), and these variabilities have an impact on the cell quality of the apheresis products used for CAR T-cell manufacturing. Hence, use of appropriate *in vitro* culture methods that promote enrichment of T_SCM_ and T_CM_ subsets within the CART product would potentially enhance their clinical efficacy. Preclinical studies in xenogeneic animal models of B-cell lymphoma have shown that CART19 cells manufactured from CD4 and CD8 T_N_ and T_CM_ cells have improved antitumor effect compared with those CART derived from more differentiated T-cell subsets (20, 72). In line with this data, studies in xenogeneic human lymphoma models have shown that CART products with a high proportion of T_SCM_ cells have enhanced *in vivo* anti-lymphoma effect compared with those with a lower proportion of T_SCM_ (73), highlighting the concept that CART products enriched with less differentiated memory T cells could contribute to improved clinical efficacy.

Development of CART products manufactured from T_N_ cells resulting in high proportion of T_SCM_ and T_CM_ cells has been hampered by the absence of methods to isolate T_N_ cells under clinical-grade manufacturing (GMP) conditions. However, recent technical advances using culture conditions with IL-7/IL-15 and the addition of IL-21 may enhance the enrichment for T_SCM_ cells in the final CART product (74–76), which can be further increased with the addition of drugs blocking T-cell differentiation, such as glycogen synthase-3 inhibitors (77). Nevertheless, robust clinical-grade protocols for generating T_SCM_-enriched CART products have not been developed so far. Recently, a few CART19 clinical trials for DLBCL have been conducted in which CART products were manufactured from CD62L^+^ isolated T cells to generate cellular products enriched for T_CM_ cells (21, 78); however, due to prolonged culture conditions, enrichment for T_SCM_ and T_CM_ subsets in the infused product could not be demonstrated. The clinical impact of this strategy is being tested in recent CART19 clinical trials with very short culture or following isolation of T_N_-T_CM_ T cells for CART manufacturing, in patients with DLBCL (NCT02153580). Combinatorial approaches like CD62L^+^ selection with the use of cytokines promoting T_SCM_ generation (e.g., IL-15 and IL-21) during the *ex vivo* expansion could further improve the enrichment of T_SCM_ and T_CM_ CAR T cells, and this strategy is already being developed in a clinical trial of CART19 for patients with DLBCL and others B-cell NHL lymphoma (NCT04653649). Overall, preclinical studies convincingly demonstrated that *in vivo* administration of less-differentiated memory T cells results in enhanced engraftment, expansion, and persistence, which are features required for clinical efficacy of CART therapy for B-cell malignancies (16, 24, 25, 41). Remarkably, these findings are being replicated in clinical studies of patients with B-cell malignancies treated with CART19.

Preliminary studies in 14 patients with B-cell lymphoma treated with CAR19 T cells have shown the existence of an association between the presence of T_SCM_ (> 5% of total T cells) in the infusion product and the *in vivo* expansion (79). The generation of products with these features was mostly obtained by substituting IL-7 and IL-15 combination with IL-2 as growth factors in the culture. Because those patients did not receive lymphodepleting chemotherapy, a correlation with clinical response could not be proven; however, this was the first clinical study showing a clear association between the infusion of less differentiated memory CAR19 T cells and expansion, one of the most clinically relevant features required to obtain a complete remission (23–25). Further confirmation of the clinical impact of administration of CART memory T cells comes from a clinical study in patients with CLL treated with CART19 (80). Patients with complete response exhibited dramatic expansion of CAR19 T cells and this was associated with the presence of T_SCM_ cells in the leukapheresis product. Moreover, clinical responses were seen mostly in those patients receiving CART19 products enriched in CD8^+^ CD27^+^ PD-1^-^ T cells with a high expression of IL-6R and STAT-3-related cytokine secretion such as IL-21, which are closely related to T_SCM_. The correlation between presence of memory T cells and clinical response to CAR-T therapy was further demonstrated in a very recent study with DLBCL patients treated with Axi-cel (81). Here, the infusion products of 24 patients with B-cell lymphoma (mostly DLBCL) were analyzed for gene expression using a transcriptome profiling approach. Patients with CRs and durable responses received CART19 products highly enriched (3-fold higher) in CCR7^+^ CD27^+^ memory CD8^+^ T cells compared with those patients with progressive disease.

Further data on the relevance of infusion product T-cell fitness come from a recent analysis of DLBCL patients treated with Axi-cel (41). In this study, the number of infused CD45RA^+^ CCR7^+^ (i.e., T_N_ and T_SCM_ cells) significantly correlated with peak CAR19 T-cell levels, a lower double timing, and durable responses; interestingly, a high number of CD8^+^ T cells were needed to promote clinically meaningful responses, at least in patients with a high TB. In contrast, CART19 products enriched in other T-cell subsets failing to express that phenotype correlated with clinical failures, highlighting the concept that CAR T-cell subsets resembling T_N_ and T_SCM_ are the most responsible for achieving a durable response.

In addition, the variability of CD4^+^ and CD8^+^ T cells in the apheresis product results in heterogeneous CART products, with a large variation in the proportion of CD4^+^ and CD8^+^ T cells, which may contribute to differences in clinical efficacy and/or toxicity. In fact, preclinical studies in xenogeneic animal models of B-cell lymphoma have shown increased antitumor effect of CAR19 T cells with a defined composition of CD4:CD8 ratios (e.g., ratio 1:1). As previously mentioned, this concept has been moved to the clinic with the development of clinical trials with CART19 products defined with a CD4:CD8 ratio of 1 in patients with DLBCL (25). Clinical efficacy of this strategy in patients with DLBCL seems to be comparable to other CART19 studies (23, 24, 34, 82), although a very low incidence of severe complications (CRS and ICANS) was noticed. Since no comparisons with other manufactured CART19 products have been done, it remains to be seen if this approach results in improved efficacy and/or toxicity in patients with DLBCL.

### Pharmacological Intervention to Enhance Generation of Memory CART19 Cells

As CART19 therapy needs T-cell expansion and appropriate engraftment, pharmacologic interventions to enhance these properties have been studied. Ibrutinib, a first-in-class irreversible inhibitor of Bruton tyrosine kinase, has shown to improve several T-cell effector functions (i.e., activation, INFγ secretion). Studies in patients with chronic lymphocytic leukemia (CLL) receiving CART19 have shown that prolonged ibrutinib treatment contributes to decreased expression of T-cell inhibitory molecules (PD-1 and CD200) with reversion of functional status of CAR19 T cells and enhanced *in vivo* proliferation (83, 84). These data prompted to evaluate ibrutinib administration before leukapheresis and concurrent with CART19 therapy. No differences in T-cell subset composition were described in these studies (83, 84), but preliminary data from a phase 1 clinical trial in patients with R/R CLL and indolent B-NHL (NCT00466531) reported significantly greater *ex vivo* expansion and proportions of CAR19 T cells expressing CD62L and CD127 in patients on ibrutinib at the time of leukapheresis (n = 5) (85). Further studies are needed to confirm these findings as well as if the effect of ibrutinib on CAR19 T cells is seen also in DLBCL patients.

CAR T-cell expansion, differentiation, and persistence results from the integration of signals arising from multiple receptors—these signals converge to activate two major signal transduction networks within T cells: the MAPK and PI3K/AKT/mTOR pathways (86). Activation of phosphoinositide 3-kinase (PI3K) has a critical effect on T-cell proliferation, survival, and effector/memory subset formation. PI3Kδ promotes mTOR signaling and increases T-cell metabolic activity, which facilitates effector T-cell differentiation and function. *Ex vivo* pharmacologic blockade of PI3K or AKT during CART19 manufacture contributed to generation of memory T cell with enhanced antitumor effect in preclinical models (86), and this strategy has been moved to the clinic in patients with multiple myeloma receiving CART directed to the BCMA antigen (87).

Pharmacological modulation of CAR T cells during manufacturing is a matter of intensive research, and several drugs are being actively studied to promote CART products enriched in less differentiated memory T cells (i.e., BET inhibitors, c-myc inhibitors) (88, 89). The clinical benefit of all these strategies in DLBCL patients receiving CART19 remains to be proven.

## Antigen Escape and Lack of Response to CART19

Antigen downregulation and antigen escape are among the main causes of relapse in patients with B-cell malignancies after CART19 therapy (90). Initial studies on this mechanism of resistance come from clinical trials of CART19 in patients with B-ALL, in which, up to 30% of the relapsing patients had CD19-negative tumors. Differences in numbers of patients suffering CD19-negative relapses also depend on the costimulation of the CAR used (4-1BB vs. CD28), with preliminary data suggesting a higher frequency in patients receiving 4-1BB-costimulated CARs (91–94).

The impact of the occurrence of CD19-negative relapses in patients with DLBCL after CART19 therapy has been less studied, in part because lymph node biopsies at the time of relapse are difficult to obtain and the techniques regularly used to analyze for CD19 expression on tumors (e.g., immunohistochemistry) are unreliable. CD19 expression at the time of relapse has not been systematically studied in pharmaceutical-sponsored clinical trials of CART19 for DLBCL patients. Estimations of 20%–30% CART19-negative relapses have been documented (90, 95) but these numbers may be underestimated since studies on CD19 expression were not done in most relapsed patients. A recent retrospective multicentric study from the US Lymphoma CAR T-cell Consortium reported a 30% of CD19-negative progression after Axi-cel treatment. Measurement of CD19 expression was assessed by flow cytometry and/or immunohistochemistry, although cutoff for CD19 positivity varied widely between centers (95). More accurate information on the occurrence of CD19 escape after CART19 would come from studies in DLBCL patients treated in academic centers with already approved CART19 therapies. Thus, a study undergone at Stanford University revealed that up to 60% of DLBCL patients treated with Axi-cel had tumors at the time of relapse with diminished or CD19-negative expression (96). Interestingly, in this study, a correlation was also found between pre-CART therapy density of expression of CD19 antigen on tumor cells, as detected by sensitive flow cytometry, and outcome. Investigators set up a threshold limit for defining low versus high expression of CD19 molecules on tumor cells and showed an increased risk of progression (50%) after Axi-cel therapy in patients with low-expression CD19 DLBCL. CD19 antigen escape also happened in patients receiving a bispecific CART. In a small study with an in-house developed tandem CD19/CD22 4-1BB-z CART for DLBCL (n=21), 4 out of 14 patients had CD19-negative tumors at relapsing while preserving CD22 antigen expression (96). It remains to be seen if other strategies targeting simultaneously CD19 combined with other B-cell antigens such as CD20 offer additional advantage related to efficacy and overcome antigen escape (97). Mechanisms explaining CD19 escape include antigen loss due to splice variants that specifically lacks the exon containing the CD19 extracellular epitope recognized by all three FDA-approved CART19 used in the clinic (98). Alternatively, CD19 variants that lack the transmembrane domain may also occur and therefore loss of surface expression. Expression of the CAR on the tumor B cell and interaction with CD19 antigen may result in masking it from recognition by the CAR T cells and therefore conferring resistance to CART19 (99). However, this mechanism, described in a single patient with ALL, has not been detected in DLBCL patients so far.

## CAR T-Cell Functionality, Inhibitory Receptor Expression, and Clinical Efficacy

The search for biological features of the infused CAR T cells predictive of outcome is hampered by the high variability of the products infused into the patients, regarding transduction efficiency, CAR expression, and T-cell subset composition. Eventually, the efficacy will depend upon the antitumor effect of the infused CAR T cells and thus functional studies of the infused CART product may reveal critical information on the clinical efficacy. Since CART19 products are comprised of a heterogeneous population of cells, single-cell analysis may provide information on relevant cells that may be associated to antitumor effect (100).

Rossi et al. studied 22 patients with B-NHL (19 had DLBCL) treated with Axi-cel and their CART19 infused products, using single cell analysis that allows the detection of more than 30 proteins secreted by a single T cell (101). They found that patients achieving a response had a proportion of CAR19 T cells (up to 25% of the product) secreting at least two of those proteins from at least two different families (i.e., inflammatory, effector, chemoattractive, stimulatory, or regulatory) compared to patients who did not respond. Interestingly, these cells called “polyfunctional T cells” were CD4^+^ but no association with a particular phenotype could be demonstrated. If this study represents a measure of the product quality or specific antitumor, T-cell subsets remain an unanswered question so far, as well as the reproducibility of these data in a larger dataset of patients (33).

T-cell exhaustion has been implicated as an additional factor that limits the efficacy of CART therapy (102). The presence of exhausted CAR T cells is characterized by dysfunctional features such as reduced cytokine secretion and limited expansion capacity, which are markers of poor antitumor function (103). The analysis of exhausted CAR T cells in the infusion product is a matter of intensive research that may allow identification of patients who eventually do not benefit from this therapy. Thus, CART19 cells expressing PD-1 combined with either TIM-3 or LAG-3 were found in high proportion in infused products of CLL patients who failed to respond compared with lower frequencies in those who attained a CR (80). Recent studies in DLBCL patients treated with Axi-cel showed an association between lack of response and infusion of CART19 products with high proportion of CD8^+^ CAR19 T cells expressing TIM-3 and LAG-3 (81). A gene-expression signature in the infused product characterized by high expression of genes related to exhaustion and activation (e.g., LAG-3, BATF transcription factor, inhibitor of DNA binding 2 -ID2-, but not PD-1) was found in products infused to patients that failed to achieve a molecular response by assessing circulating plasma-derived cell-free DNA (81). Interestingly, a high proportion of LAG-3^+^ T cells were found in a small subset of DLBCL patients unresponsive to Tisa-cel, highlighting the potential impact of this T-cell subset and clinical response (19).

Overall, these preliminary studies show that, in addition to defined T-cell subsets, the presence of functional T-cell populations without exhaustion receptor expression characterizes CART19 products with improved clinical efficacy in DLBCL patients. However, studies on the infusion of anti–PD-1 antibodies either in combination or after CART19 failure have not resulted in significant durable responses in patients with DLBCL (104, 105).

**Figure 2:**
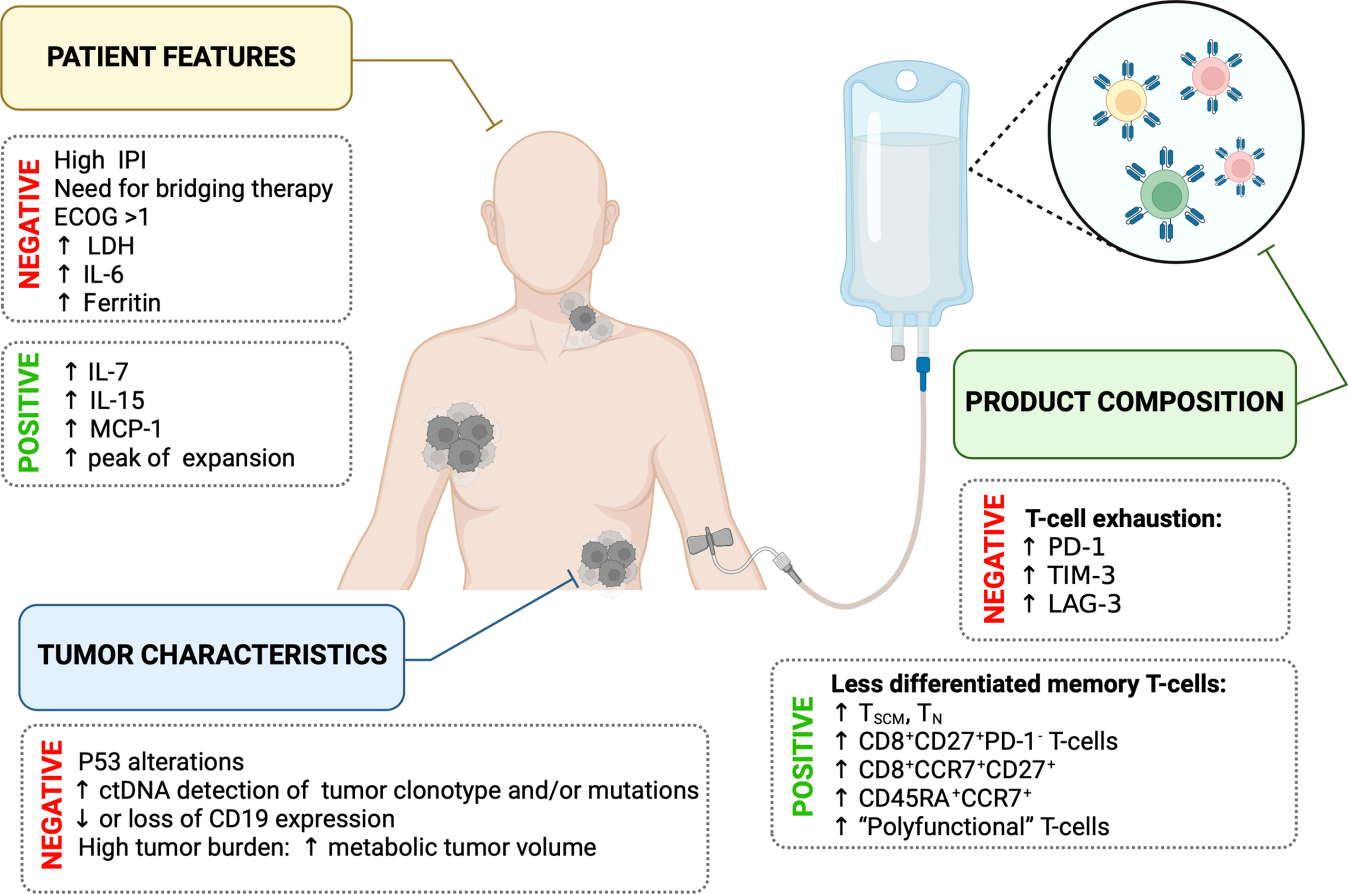
Summary of main factors influencing the efficacy of CART19 therapy.

In summary, the efficacy of CART19 therapy and subsequent clinical outcome in DLBCL are influenced by patient’s features, tumor characteristics, and the composition of the T-cell product (**Figure 2**). In-depth knowledge of each factor for every patient is highly needed to identify those patients with a high risk of treatment failure.

## Influence of Gut Microbiome on CART19 Efficacy

The microbial cells that colonize the human body, including mucosal and skin environments, are at least as abundant as our somatic cells and certainly contain far more genes than our human genome. Their gene diversity encodes outstanding mechanism and metabolic competences that influence their own microbial niche, host tissue specific, and immune-cells function (106). Ninety-nine percent of the entire microbial mass is within the gastrointestinal tract, and it exerts both local and long-distance effects and the bacterial community varies between luminal and mucosa-associated communities. Studies in germ-free animals have revealed evidence for tumor-promoting effects of the microbiota in various organs, including the skin, colon, liver, breast, and lungs. On the other hand, intestinal microbiota can modulate the antitumor response through activation of innate immunity, which may convert tumor tolerance into enhanced antitumor immune responses (107).

Several preclinical and clinical studies proved that intestinal microbiota could modulate antitumor effect of chemotherapy. Thus, Cy promoted the translocation of distinct Gram^+^ bacteria (mainly *Lactobacillus johnsonii* and *Enterococcus hirae*) that elicited effector T_H_17 cell responses associated with tumor control (108). *Enterococcus hirae* translocated from the small intestine to secondary lymphoid organs increased intratumoral CD8^+^/Treg ratio (109). These are interesting data as Cy is used as conditioning treatment in CART19 therapy and can induce microbial translocation that can amplify effector T-cell function (110). Retrospective studies analyzed microbiome in patients receiving allogeneic hematopoietic cell transplantation (allo-HSCT) and found significant differences from healthy volunteers. Profiling of intestinal microbiota in those patients revealed that a higher diversity of the intestinal microbiome was associated with lower graft-versus-host disease-related mortality (111).

A recent study investigated whether intestinal microbiome could modulate CAR19 T-cell activity and subsequent clinical outcomes in patients with B-NHL and B-ALL (112). Antibiotic exposure during the four-week period before CART19 treatment was correlated with reduced survival. Specifically, exposure to piperacillin/tazobactam, meropenem, and imipenem/cilastatin was associated with worse OS and PFS as well as higher proportion of ICANS, independently of the CART19 costimulatory domain (e.g., CD28 or 4-1BB). In addition, it was found that compositions of fecal samples from recipients of CART19 therapy were significantly different from healthy volunteers, represented by a lower diversity. Furthermore, they described that a higher relative abundance of selected microbial taxa, including *Ruminococcus*, *Bacteroides*, and *Faecalibacterium*, was associated with day 100 complete response. These bacterial taxa and *Enterococcus* have been associated with improved response and reduced toxicity to immune checkpoint blockade therapy (113, 114), as well as immune cell dynamics after allo-HSCT (115). Further studies are needed to confirm these findings and to understand the mechanisms by which bacterial taxa and bacterial metabolites influence the immune system to improve patient outcomes after CART therapy.

## Treatment Options After CART19 Failure

Significant numbers of R/R DLBCL patients will experience disease progression following CART19, but little data are available on outcomes of these patients. Initial studies have reported that about 75% of DLBCL patients progressing after CART19 would be candidates for salvage therapy, while the remaining 25% would receive palliative treatment only (95, 116). Current data show a very poor outcome for those patients refractory to CART19. Thus, a recent multicenter retrospective study of the US Lymphoma CAR T-cell Consortium showed a median PFS of 55 days from first therapy after Axi-cel failure (95). Furthermore, these studies have shown that CART19-refractory patients or those with progression within 30 days of CAR19 T-cell infusion have a dismal outcome (116). Nevertheless, no differences in efficacy or DOR were reported among different current salvage therapies post-CART19 (95, 117).

CART19 failure represents a complex and challenging scenario, since most available salvage strategies, including conventional and novel therapies, have been approved based on clinical trials that specifically excluded patients receiving CART19 therapy. While priority should be given to inclusion of patients in clinical trials, conventional therapy may still provide short-term disease control in a limited number of patients. Number of prior lines and previous refractoriness to chemotherapy should be carefully evaluated before choosing the salvage therapy, and allogeneic hematopoietic transplantation should be considered if a response has been achieved.

**Figure 3:**
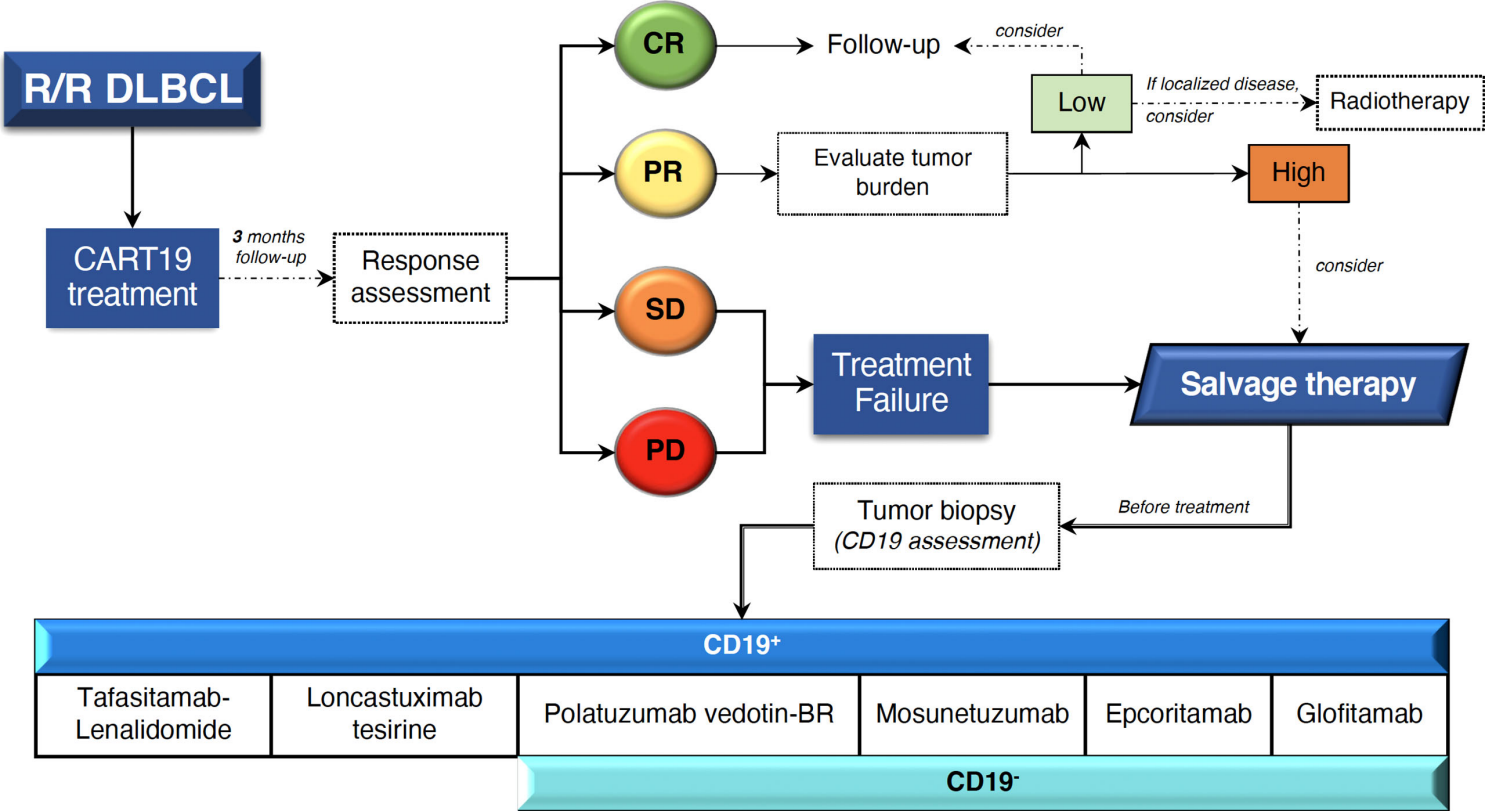
Management proposal for patients with R/R DLBCL who fail CART19 treatment. Novel clinically relevant therapies based on CD19 tumor expression are shown. CR, complete response; PR, partial response; SD, stable disease; PD, progressive disease; BR, bendamustine-rituximab.

New treatment approaches include monoclonal antibodies (mAbs), antibody-drug conjugates, bispecific antibodies (bsAbs), immunomodulatory drugs, checkpoint inhibitors, molecular pathway inhibitors, and epigenetic-modifying molecules. Current therapeutic options that should be considered for the treatment of patients who have failed to CART19 are reviewed below (**Figure 3**), and main toxicities are listed in **Table 2**.

**Table 2:** Therapeutic Options After CART Failure: Safety Profile.

AGENT	DESIGN	SCHEME	SIDE EFFECTS
**Tafasitamab**	**mAb** 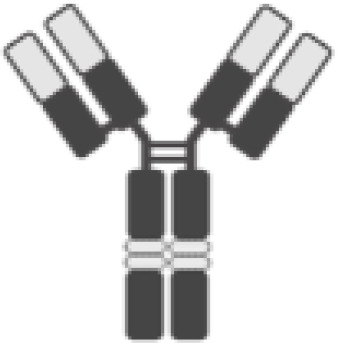 **CD19**	Tafasitamab-Lenalidomide	***Any grade:*** Rash (27%), diarrhea (32%). ***Grade ≥3:*** Neutropenia (48%), thrombocytopenia (17%), and febrile neutropenia (12%). **SAEs:** pneumonia (6%), febrile neutropenia (6%), pulmonary embolism (4%), bronchitis (2%), atrial fibrillation (2%), and congestive cardiac failure (2%).
**Polatuzumab-vedotin**	**ACD** 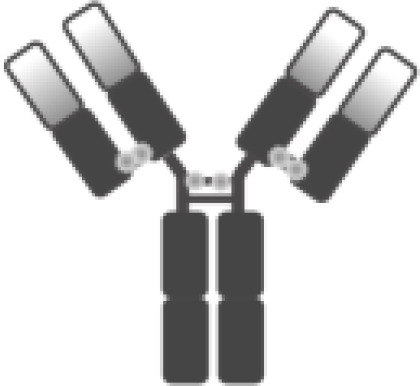 **CD79b**	Pola-BR	***Any grade:*** Peripheral neuropathy grade 1–2 (43-6%), infections (23%). ***Grade ≥3:*** Neutropenia (46.2%), thrombocytopenia (41%), increased GGT (17%), and febrile neutropenia (10.3%). Three ***fatal AEs*** (7.6%): pneumonia, hemoptysis, and pulmonary edema.
**Loncastuximab tesirine**	**ACD** 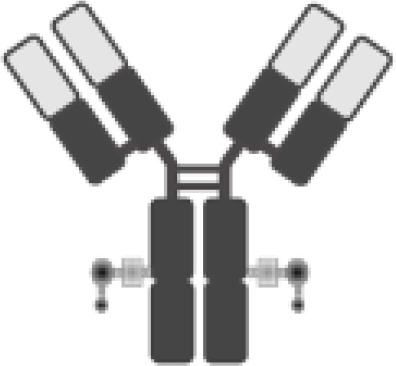 **CD19**	Single agent	***Any grade:*** nausea (23%), Peripheral edema (19%) ***Grade ≥3:*** Neutropenia (26%), thrombocytopenia (18%), and febrile neutropenia (3%). ***Fatal AEs*** (6%): sepsis, small intestinal perforation, pneumonia, and acute kidney injury.
**Glofitamab**	**bsAb** 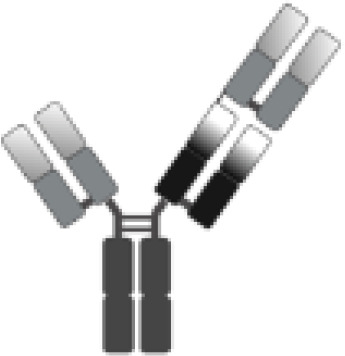 **CD20 – CD3**	Single agent	***Grade ≥3:*** Neutropenia (25.1%), thrombocytopenia (8.2%), and febrile neutropenia (2.9%). ***CRS*** * (50.3%): 46.7% grades 1–2 and 3.5% grade 3 or 4. ***Neurologic AEs*** (43.3%), ICANS**-like (5.3%). ***Fatal AEs*** (1.2%): GI hemorrhage and septic shock.
**Mosunetuzumab**	**bsAb** 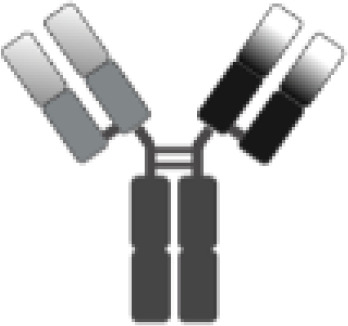 **CD20 – CD3**	Single agent	***Grade ≥3:*** Neutropenia (25.4%), hypophosphatemia (15.2%), anemia (9.1%), and febrile neutropenia (3.6%). CRS* (27.4%): 26.4% grades 1–2 and 1% grade 3. Grade 3 ***neurologic AEs*** (4.1%). ***Fatal AEs*** (1.5%): hemophagocytic lymphohistiocytosis, sepsis, candida sepsis, and pneumonia.
**Epcoritamab**	**bsAb** 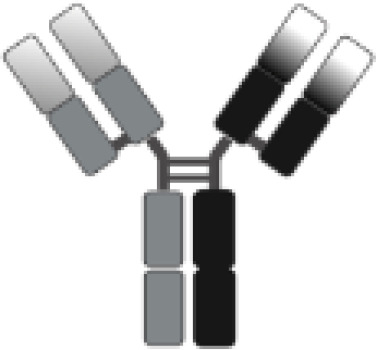 **CD20-CD3**	Single agent	Injection site reaction (47%): grades 1–2. ***Grade ≥3***: pneumonia (12%), dyspnea (6%). CRS* (59%) only grades 1–2. No discontinuations occurred due to treatment-related AEs or treatment-related deaths.

*Cytokine release syndrome **Immune effector cells-associated neurotoxicity syndrome.

### Monoclonal Antibodies

mAbs exert their antitumor capacity through several mechanisms, including complement-dependent cytotoxicity, antibody-dependent cellular cytotoxicity, and antibody-dependent cell-mediated phagocytosis commonly regulated *via* interaction between the Fc antibody chain and Fcγ receptors on immune effector cells: natural killer cells, macrophages, and γδ T cells (118).

*Tafasitamab* is an Fc-engineered, humanized, CD19 mAb with the introduction of S239D and I332E amino acid substitutions that leads to an enhancement of antigen-dependent cell-mediated cytotoxicity and antigen-dependent cell-mediated phagocytosis compared with the unmodified parental immunoglobulin G1 CD19 antibody (119). Antitumor activity of Tafasitamab against R/R B-NHL was investigated in a phase 2a study. Tafasitamab was administered as an intravenous infusion on days 1, 8, 15, and 22 of a 28-day cycle, for two cycles. Patients achieving PR or CR could continue to receive Tafasitamab until disease progression or unacceptable toxicity. Twenty-six percent of patients with R/R DLBLC responded and 6% accomplished CR. The median PFS was 2.7 months (119). To enhance the antitumor effect, a second phase 2 trial tested a potentially synergistic combination: Tafasitamab-lenalidomide. The combination was evaluated in R/R DLBCL patients who were considered ineligible to receive high-dose chemotherapy followed by ASCT. Median lines of treatment before Tafasitamab-lenalidomide were 2 (range: 1–4). Patients received intravenous Tafasitamab (days 1, 8, 15, and 22) simultaneously with oral lenalidomide (days 1–21) for up to 12 cycles (28 days each), followed by Tafasitamab monotherapy (in patients with stable disease or better) until disease progression (120). After >35 months follow-up, ORR was 57.5% including 40% of patients achieving CR. The median PFS was 11.6 months. Patients treated with CART19 were not included (121). Interestingly, remission of DLBCL after CART19 was reported in one patient who previously failed to Tafasitamab. CD19-targeted therapies could be effective despite being administered sequentially (122).

### Antibody-Drug Conjugates

Antibody-drug conjugates are mAbs conjugated to cytotoxic agents. This treatment modality takes advantage of the antibody specificity for the target antigen and achieves antitumor activity through the addition of different effector molecules that induce cell death after antibody binding and internalization. Such effector molecules include cytotoxic agents (antibody-drug conjugate, ADC), bacterial or plant protein toxins (immunotoxins), and radiopharmaceutical agents (radiolabeled mAb) (123).

*Polatuzumab-vedotin* (Pola) is an ADC that consists of CD79b-binding mAb conjugated to an antimitotic agent: monomethyl auristatin E (MMAE) (124). Polatuzumab-vedotin selectively targets B cells into which MMAE is internalized and cleaved from its linker by lysosomal proteases before binding to microtubules to inhibit cell division and induce apoptosis (125). Polatuzumab vedotin plus bendamustine and rituximab (BR) received regulatory approvals for transplantation-ineligible R/R DLBCL based on primary results from a phase 1b/2 study comparing Pola-BR versus BR alone. In this scenario, Pola-BR has shown to improve CR rate (40% vs. 17.5%) and PFS (median 9.5 vs. 3.7 months) (126). Results from an extension cohort confirmed significant survival benefit with Pola-BR, but a lower ORR 41.5% and similar CR rate 38.7%. This trial excluded patients treated with CART19 therapy (127). German experience described Pola-BR and other combinations (Pola-B, Pola-R-CHP, Pola-R-Gemcitabine, Pola-R), showing ORR 48% and 14.8% of CRs in patients with R/R DLBCL (124). On another hand, Greek experienced reported ORR of 43% and 25% of CRs in patients with B-NHL, including mainly R/R DLBCL but also MCL, PMBCL, and transformed FL (128). Unfortunately, CART19 treated patients was barely represented in those studies (124, 128).

*Coltuximab ravtansine* is an ADC consisting of a humanized IgG1 anti-CD19 mAb (SAR3419) conjugated to DM4, a potent antimitotic agent that inhibits tubulin polymerization and microtubule assembly. After binding to the cell-surface antigen, the complex immunoconjugate antigen is internalized and undergoes lysosomal degradation, generating an intermediate unstable metabolite (Lysine-SPDB-DM4) that undergoes additional intracellular processing to produce DM4 (129). Modest efficacy was displayed by Coltuximab-ravtansine in a Phase 2 clinical trial for the treatment of R/R DLBCL, with ORR 43% and CR rate 14.6%. The study population has previously received a median of two treatments excluding CART19 (130).

*Loncastuximab tesirine* is an ADC comprising a humanized IgG1 anti- CD19 antibody (RB4v1.2) conjugated to SG3199, a pyrrolobenzodiazepine (PDB) dimer toxin. PDB dimers are sequence-selective DNA cross-linking agents that do not cause distortion of the DNA structure. Less DNA distortion may hide PBD dimers from DNA repair mechanisms and appears to help in maintaining their biological activity and persistence in cells (131, 132). A multicenter, single-arm phase 2 trial in patients with R/R DLBCL (median previous lines of treatment: 3; range: 2–4) was designed to determine Loncastuximab tesirine efficacy. Reported ORR was 48.3% with 24.1% of CRs and a median PFS of 4.9 months. Nine percent of patients received CART19 therapy prior to Loncastuximab tesirine, all of them with a biopsy-proven CD19-tumor expression; ORR was similar to that of the overall study population (132, 133). The median time between CART19 failure and ADC treatment was 7 months (range: 45–400 days), and in 77% of cases was administered as first therapy post-CART19. ORR was 46.2% and 15.4% achieved CR (134).

*Brentuximab vedotin* (BV) is an ADC constituted by an anti-CD30 mAb (SGN-30) conjugates to MMAE (135). BV has been approved for classical Hodgkin lymphoma, primary cutaneous anaplastic large cell lymphoma, and systemic anaplastic large-cell lymphoma (136). CD30 protein is expressed in 14% to 25% of DLBCL patients depending on the cutoff to assign positivity (136–138). BV has shown antitumor activity in patients with R/R DLBCL [median previous lines of treatment: 3 (range: 1–6)] in a phase 2 study with ORR of 44% and 17% CR rate. Interestingly, no statistical correlation between response and level of CD30 expression was found, since 12% of patients with little to no CD30, determined by visual immunohistochemistry, achieved a CR (136, 139). Patients treated with CART19 were not included in the study, as it was not yet available as a SOC (136).

*Pinatuzumab vedotin* (Pina) is an ADC composed by a humanized anti-CD22 monoclonal IgG1 antibody (MCDT2219A) with MMAE, linked to the reduced cysteines of the antibody *via* a protease cleavable linker, maleimidocaproyl-valine-citrulline-*p*-aminobenzoyloxycarbonyl (MC-vc-PAB) (140, 141). The efficacy of Pinatuzumab vedotin was evaluated in combination with Rituximab for the treatment of R/R DLBCL [median previous lines of treatment: 3 (range: 1–3)] in a Phase 2 open-label, randomized clinical trial comparing Pola-R versus Pina-R. Pina-R achieved 60% of ORR and 26% of CRs. Meanwhile, Pola-R reached a 54% ORR and 21%. CR rate. Median DOR was shown to be longer for R-Pola than R-Pina (13.4 versus 6.2 months, respectively). No correlation between CD22 expression and tumor shrinkage for Pina was observed. The inclusion of patients previously treated with CART therapy was not reported. Data derived from this study suggest that R-Pina and R-Pola may have antitumor effect against R/R DLBCL but short-term disease control (142, 143).

### Bispecific Antibodies

The term “bispecific antibody” refers to an antibody or an antibody‐derived protein construct that has binding specificities for two different antigens. bsAbs combine two different monospecific antigen‐binding regions, or variable regions, from different antibodies to achieve a single antibody or antibody‐derived molecule with bispecific antigen binding (144). bsAbs can be classified according to the presence or absence of an Fc region. bsAbs that include an Fc region can be further divided into those that exhibit a structure resembling that of an IgG molecule and those that contain additional binding sites. Different configurations of bsAbs allow modulation of valency, size, flexibility, pharmacokinetic, and pharmacodynamic properties (145).

Typically, bsAbs consist of a T‐cell receptor‐specific mAb or mAb‐derived fragment that is able to activate and expand resting T cells fused to a second mAb or mAb fragment directed against a tumor target antigen. Thus, bsAbs induce attached T cells specific cytotoxicity effect against tumor target cells without the requirement of MHC‐mediated antigen presentation (144).

*Blinatumomab* (Blina) comprises an anti-CD19 scFv linked through a linker to an anti-CD3 scFv. Blina is a bispecific T-cell engaging (BiTE) antibody construct. Blina molecules are small and rapidly cleared from circulation with a terminal half-life of 1.25 h requiring continuous intravenous infusion (IV) (146, 147). A phase 2, single agent clinical trial evaluated the use of Blina against R/R DLBCL [median previous lines of treatment: 3 (range: 1–7)] showing modest efficacy (ORR 43% and CR rate 19%) (146). Another phase 2 study evaluated the efficacy of Blina as second salvage for R/R DLBCL and found a 37% ORR with 22% CRs and a median PFS of 2.5 months (148). These studies did not include patients treated with CART19.

*Glofitamab* is a full-length bsAb possessing a 2:1 structure with bivalency for CD20 on B cells and monovalency for CD3 on T cells. CD20 bivalency preserves this potency in the presence of competing anti-CD20 antibodies, providing the opportunity for pre or cotreatment with these agents. Patients with R/R B-NHL, including DLBCL, tFL, or other aggressive histology, were treated with Glofitamab alone as part of a phase 1 dose-escalation study. Patients had a median of 3 (range: 1–13) prior lines of therapy. A single dose of Obinutuzumab was administered 7 days before treatment with Glofitamab to reduce TB and expected toxicity. Glofitamab has longer half-life compared with BiTES (non-Fc), allowing it to be administered every 14 or 21 days. Patients previously treated with CART19 were poorly represented in this study. Across all doses, ORR was 53.8% and CR rate was 36.8%. At the recommended dose, ORR and CR were 71.4% and 64.3%, respectively (149). Furthermore, preliminary data from the use of Glofitamab in combination with Polatuzumab vedotin for the treatment of R/R B-NHL reported ORR of 73% and 51.5% of CRs. With a short follow-up and small number of treated patients, these encouraging data need to be confirmed in larger trials (150).

*Mosunetuzumab* is a full-length, humanized, IgG1–based bsAb targeting CD20 and CD3 (151). A phase 1/1b dose-escalation and expansion study evaluated Mosunetuzumab for the treatment of patients with R/R DLBCL. The study population included 9.6% of patients who had received prior CART19 treatment. Mosunetuzumab was administered intravenously as low and intermediate step-up doses on days 1 and 8 of cycle 1, with the target dose on day 15 and on day 1 of subsequent 21-day cycles. ORR was 34.9% and CR rate was 19.4%. In the post-CART19 therapy group (n = 15), ORR was 36.8% and CR was 26.3%, but DOR was not shown in this particular subgroup of patients (152).

*Odronextamab* (REGN1979) is a first-in-class, hinge-stabilized, fully human IgG4 bsAb directed against CD20 and CD3. A phase 1 clinical trial was developed to evaluate efficacy of Odronextamab for the treatment of R/R B-NHL including R/R DLBCL. Odronextamab is administered in a step-up dosing regimen and dexamethasone was administered as premedication to reduce expected toxicities. Preliminary results reported that, among R/R DLBCL patients, 37% achieved some response and 26% had CR. In those DLBCL patients who relapsed after CART therapy, 31% responded, with 22% CRs (153).

*Epcoritamab* is a full-length human IgG1 bsAb recognizing CD3 and CD20, generated by a new method, using controlled Fab-arm exchange of mAb half-molecules. It was designed to offer a longer plasma half-life compared to others bsAbs. Fc-mediated effector functions were silenced by the introduction of three amino acid mutations in the Fc region (154). Safety and efficacy of Epcoritamab were assessed in a phase 1/2 clinical trial to treat R/R B-NHL patients, including R/R DLBCL. In contrast to previous bsAbs, Epcoritamab administration is subcutaneous (SC). A priming SC dose was given on day 1 of cycle 1 and an intermediate dose on day 8 of cycle 1, followed by full doses administered in 28-day cycles until disease progression or unacceptable toxicity. Eleven percent of patients included in the study had failed CART19 therapy. ORR was 68%, with 38% of them achieving CR. Among four patients with R/R DLBCL who had received CART19 before epcoritamab, all of them responded with two of them achieving a CR (155).

### Immune Checkpoint Inhibitors

High baseline levels of inhibitory checkpoint protein expression (PD-L1, LAG3, and TIM3) by tumor and tumor microenvironment cells have been reported in patients who do not respond to CAR T cells (19, 24). A phase 1/2a study evaluated the efficacy of *Pembrolizumab* (an anti–PD-1 immune checkpoint inhibitor) in patients with B-NHL after failure to CART19 therapy. A small number of patients were treated (n = 12) with an ORR of 25% and 8% CR (n = 1). Immune profiling revealed increased CAR T-cell activation and proliferation and less T-cell exhaustion in clinical responders (104). Furthermore, results of another small study revealed a higher PD-1/PD-L1 expression in responsive patients with anti–PD-1 therapy as compared to that in non-responders (156). Safety and efficacy of anti–PD-1 administration early after CAR19 T-cell infusion are being evaluated in clinical trials, with initial data showing 91% ORR and 64% CR after Liso-cel and *Durvalumab* combination (157).

Moreover, efficacy of the combination of an anti-CD20 mAb, *atezolizumab* (anti-PDL1) and Polatuzumab vedotin for the treatment of R/R DLBCL was evaluated in a phase 1b study. This clinical trial revealed a 13% CR rate, but significant toxicities, with 24% of grade 3–5 adverse events and 10% of serious adverse events. Based on safety issues and the limited efficacy, no further development of that triplet combination was pursued (158).

### Molecular Pathway Inhibitors

Exportin 1 (XPO1), one of eight nucleo-cytoplasmic shuttling proteins involved in the export of proteins from the nucleus to the cytoplasm, is overexpressed in DLBCL. XPO1 blockade in DLBCL re-establishes the growth regulating effects of multiple tumor suppressor proteins by forcing their nuclear retention and potentially reverses chemotherapy resistance. *Selinexor*, an oral selective inhibitor of XPO1, was studied in transplant-ineligible R/R DLBCL (2–5 previous lines of treatment) in the open-label, single-arm SADAL phase 2b trial. ORR was found to be 28% with 12% CR rate and a median PFS of 2.6 months only (159).

### Allogeneic Hematopoietic Cell Transplantation

Allo-HSCT remains an SOC for DLBCL patients with chemosensitive relapse after prior ASCT (160). Allo-HSCT registry studies on DLBCL patients relapsing after ASCT consistently showed 3-year PFS rates of 30% to 40%. Allo-HSCT is a potentially curative cellular immunotherapy for R/R DLBCL and should be considered as a therapeutic option in this setting for eligible patients. Another potential field of research for allo-HSCT might be pre-emptive treatment of incomplete responses to CAR19 T cells (161). Recently, a series of patients receiving allo-HSCT after CART19 have reported a 2-year PFS of 30% and OS of 40%, although non-relapse mortality was as high as 25% (162).

## Conclusions

Following the designation as a breakthrough treatment, CART therapy had an exponential growth in basic and clinical research in the field. Hematological malignancies and patients with B-cell tumors in particular have significant clinical benefit from this treatment. However, 5 years from the first FDA-approval CART therapy, it is becoming evident the limitations of this therapy, and further improvements are needed to allow translation of a clinical benefit to a significant proportion of patients that are actually unresponsive. Preclinical and clinical CART research has advanced rapidly to identify biological and clinical factors associated with response. In contrast to other therapies, tumor biological features are not the most critically involved determining a response to CART therapy. Other non-tumor specific factors such as CART production technologies, T-cell subsets composition, T-cell features within the “starting material”, functional state of pre-infusion CAR T cells (i.e., activation patterns and expression of inhibitory receptors), in addition to *in vivo* expansion and persistence of CAR T cells, have been associated in different clinical trials as critical determinants of response. Further validation and applicability of some of these studies will potentially allow to identifying patients with higher chances of benefit from CART therapy. The prognosis of relapse/refractory patients not responding to CART therapy is extremely poor, and this situation represents an unmet medical need. Significant efforts are being made to identify new therapies that may rescue patients in that situation. Recent clinical trials with new drugs, including bispecific antibodies, drug-conjugated antibodies, CART targeting antigens other tan CD19, and allogeneic hematopoietic transplantation, would open a new future for patients in the post-CART19 era.

## Author Contributions

AC and JB performed the literature search and conducted extraction of data from relevant studies. AC, JB, CA-F, and LE-G critically reviewed the literature search. AC, JB, CA-F, and LE-G wrote and revised the manuscript, figures, references, and tables. All authors contributed to the article and approved the submitted version.

## Funding

This work was supported in part by grants from La Marató TV3 (Exp. 20130710), Deutsche José Carreras Leukämie Stiftung (DJCSL 10R/2016), Beca Carlos Antonio López (Gobierno paraguayo), Instituto de Salud Carlos III (PI15/1383 and PI18/01023), Fondos FEDER Fundación Bancaria ‘La Caixa’, Ministerio de Economía y Competitividad (RETOS; RTC 2015-3393-1) and AGAUR (2017SGR1395).

## Conflict of Interest

The authors declare that the research was conducted in the absence of any commercial or financial relationships that could be construed as a potential conflict of interest.

## Publisher’s Note

All claims expressed in this article are solely those of the authors and do not necessarily represent those of their affiliated organizations, or those of the publisher, the editors and the reviewers. Any product that may be evaluated in this article, or claim that may be made by its manufacturer, is not guaranteed or endorsed by the publisher.
